# Proapoptotic Role of Potassium Ions in Liver Cells

**DOI:** 10.1155/2016/1729135

**Published:** 2016-03-16

**Authors:** Zhenglin Xia, Xusen Huang, Kaiyun Chen, Hanning Wang, Jinfeng Xiao, Ke He, Rui Huang, Xiaopeng Duan, Hao Liu, Jinqian Zhang, Guoan Xiang

**Affiliations:** ^1^Third Clinical Medical School of Southern Medical University, Guangzhou 510515, China; ^2^Department of General Surgery, The Second People's Hospital of Guangdong Province, Southern Medical University, Guangzhou 510515, China; ^3^Department of Gastrointestinal Surgery, The Affiliated Hospital, Youjiang Medical University for Nationalities, Guangxi 533000, China; ^4^Department of Vascular Surgery, Nanfang Hospital, Southern Medical University, Guangzhou 510515, China; ^5^Laboratory Medicine, The Second People's Hospital of Guangdong Province, Southern Medical University, Guangzhou 510515, China

## Abstract

Potassium channels are transmembrane proteins that selectively promote the infiltration of potassium ions. The significance of these channels for tumor biology has become obvious. However, the effects of potassium ions on the tumor or normal cells have seldom been studied. To address this problem, we studied the biological effects of L02 and HepG2 cells with ectogenous potassium ions. Cell proliferation, cell cycle, and apoptosis rate were analyzed. Our results indicated that potassium ions inhibited proliferation of L02 and HepG2 cells and promoted their apoptosis. Potassium ions induced apoptosis through regulating Bcl-2 family members and depolarized the mitochondrial membrane, especially for HepG2 cell. These biological effects were associated with channel protein HERG. By facilitating expression of channel protein HERG, potassium ions may prevent it from being shunted to procancerous pathways by inducing apoptosis. These results demonstrated that potassium ions may be a key regulator of liver cell function. Thus, our findings suggest that potassium ions could inhibit tumorigenesis through inducing apoptosis of hepatoma cells by upregulating potassium ions transport channel proteins HERG and VDAC1.

## 1. Introduction

The plasma membrane (PM) ion channels involve almost all of the basic cellular processes and the malignant phenotype of tumor cells. Ion fluxes regulate cell volume and membrane potential through their ion channels and participate in intracellular signal transduction and controlling cell functions. Moreover, in the process of tumorigenesis development, the differences on tumor gene expression levels are determined by ion channels, which may involve, at least in part, a number of pathophysiological features associated with malignant growth [1–3].

In the ion transport molecular family, based on the biochemical structure and highest variability, potassium channels might be the most likely ones to be designed for the targeted therapy of the channel in cancer [4]. It could be used as a new research direction, providing important clues in the development of new therapeutic agents [5]. Thus, the study of ion channel serving as a new target for the diagnosis and treatment of cancer is very important. In this study, we compared the effect of potassium ions in L02 and HepG2 cells and investigated the regulation mechanism of cell functional changes induced by potassium ions.

The differential expressions of potassium channels are frequently observed in different tumors; these differences make tumors have many advantages in biological behaviors [6, 7]. Expression changes are seen in the genome, transcription, translation, or epigenetic level and can also adjust the expression level of potassium channel through the upstream changes in some cases [8, 9]. Some hormones or growth factors can activate potassium channels and cause abnormal gene expressions of potassium channels [10]. The changes of cell death, proliferation, adhesion, and migration have a significant impact on life activities. All these changes can affect the tumorigenesis. Therefore, interruption of the expression of potassium channels combined with current treatment may significantly improve the treatment of cancer. In short, interfering with potassium channel expression or activity may offer a new therapy for liver cancer [4].

## 2. Materials and Methods

### 2.1. Preparation of Plates Coated with Potassium Ions

PBS with different concentrations of potassium ions was prepared and the abbreviations represent K 0 (0 mmol/L), K 25 (3.75 mmol/L), K 50 (7.5 mmol/L), K 75 (11.25 mmol/L), and K 100 (15 mmol/L). The dispersed PBS were added to 6-well plates (add 200 *μ*L per well) or 96-well plates (add 10 *μ*L per well) and then dried at 100°C for 2 hours in air, sterilized by ultraviolet irradiation for half an hour.

### 2.2. Cell Culture and Treatment

Hepatic cells line L02 cell and hepatoma cells line HepG2 cell were cocultured with different concentrations of potassium ions and cultured in DMEM supplemented with 10% FBS (Gibco, Carlsbad, CA, USA) in 5% CO_2_ at 37°C.

### 2.3. Cell Proliferation and Viability

Cell counting kit was purchased from DOJINDO (EQ645; Kumamoto, Japan) to determine cell proliferation. To conduct the assay, L02 (3 × 10^3^) and HepG2 (3 × 10^3^) cells cultured in 96-well plates were treated using the above methods. 10 *μ*L CCK-8 solutions were added to each well. Cells were incubated at 37°C incubator for 1 hr. A microplate reader (Thermo Scientific, Waltham, PA, USA) was used to detect the absorbance values at 450 nm. Each group consists of five replicates. The data was repeated in five independent experiments and the proliferation of cells was observed at different time points as indicated.

For cell counting, L02 (3 × 10^5^) and HepG2 cells (3 × 10^5^) were added to 6-well plates treated by the above methods with 2 mL of growth medium, and then the cells were observed according to the general protocol by optical microscope. The total cellular scores were counted by cell counter plate after 48 hrs.

### 2.4. Annexin V-FITC/7-AAD Staining Assay

L02 and HepG2 cells were treated with potassium ions as described in the above method in 6-well plates. The cells were then collected and washed twice with PBS. After centrifugation, cells were resuspended in 100 *μ*L of binding buffer and then stained by Annexin V-FITC/7-AAD (640906/420404; BioLegend, CA, USA) for analysis of the apoptosis of cells.

### 2.5. Cells Cycle Analysis

L02 and HepG2 cells were cultivated in 6-well plates treated by the above methods and then cultured for 48 hrs. The cells were collected, fixed with 70% ethanol overnight and then resuspended in cold PBS, and then incubated with 1 mg/mL 7-AAD (420404; BioLegend, CA, USA) at 37°C for 15 min. The samples were detected by using FACScalibur flow cytometer (BD Biosciences, San Diego, CA, USA), and the proportions of cells in the G1, S, and G2 phases were investigated by using ModFit LTTM software (BD Biosciences, San Diego, USA).

### 2.6. Western Blotting and Antibodies

The total protein concentration was detected using the Pierce BCA assay (23225; Thermo Scientific, Waltham, PA, USA) and a microplate reader (Thermo Scientific, Waltham, PA, USA) at 562 nm. Cell lysates were resolved in 10% SDS-PAGE and transferred onto a PVDF membrane (ISEQ00010; Millipore, MA, USA). Target proteins were detected by using specific antibodies. The primary antibodies were used in a 1 : 1500 dilution. The bands were investigated using the enhanced chemiluminescence system (32209; Thermo Scientific, Waltham, PA, USA). All the data were quantified by using Bio-Profil BiolD software (S:11.640150; VILBER, France). The primary antibodies are anti-Bax (sc-493; Santa Cruz, TX, USA), anti-caspase-3 (sc-7148; Santa Cruz, TX, USA), anti-P53 (sc-126; Santa Cruz, TX, USA), anti-HERG (sc-48428; Santa Cruz, TX, USA), anti-VDAC1/Porin antibody (ab15895; Abcam, Cambridge, MA), anti-ACSS1 (ab69270; Abcam, Cambridge, MA), anti-Bcl-2 (2870; CST, Danvers, MA, USA), and anti-GAPDH (5174; CST, Danvers, MA, USA).

### 2.7. Mitochondrial Membrane Depolarization

JC-10 (Enzo Life Sciences, New York, USA) was used to detect the mitochondrial membrane depolarization of cells. The absorbing value of JC-10 lies on the polarization level of mitochondrial membrane. Hyperpolarized mitochondria was complicated with higher absorbent of JC-10 compared to depolarization. After treatment, L02 and HepG2 cells were washed once with PBS and then incubated with 500 *μ*L of 1x JC-10 dye-loading solution for 40 minutes. After incubation, the cells were subjected to flow cytometry.

### 2.8. Caspase-3/7 Activity Assay

L02 and HepG2 cells were cultured in 96-well plates treated by the above methods and incubated for 48 hrs. After the treatment with potassium ion, caspase-3/7 activity in liver cells was analyzed using caspase-Glo 3/7 assay kit.

### 2.9. Statistics

All data were showed as mean ± SD, from at least three averaged replicates of independent experiments. Statistical comparison of quantitative data in the group was determined by one way ANOVA or Student's *t*-test. To determine differences between groups not normally distributed, medians were compared using Kruskal-Wallis analysis of variance. SPSS 19.0 software (SPSS Inc., Chicago, USA) was used for data analysis. *P* < 0.05 was regarded as statistically significant.

## 3. Results

### 3.1. The Potassium Ions Inhibited Cell Proliferation in L02 and HepG2 Cells

**Figure 1:**
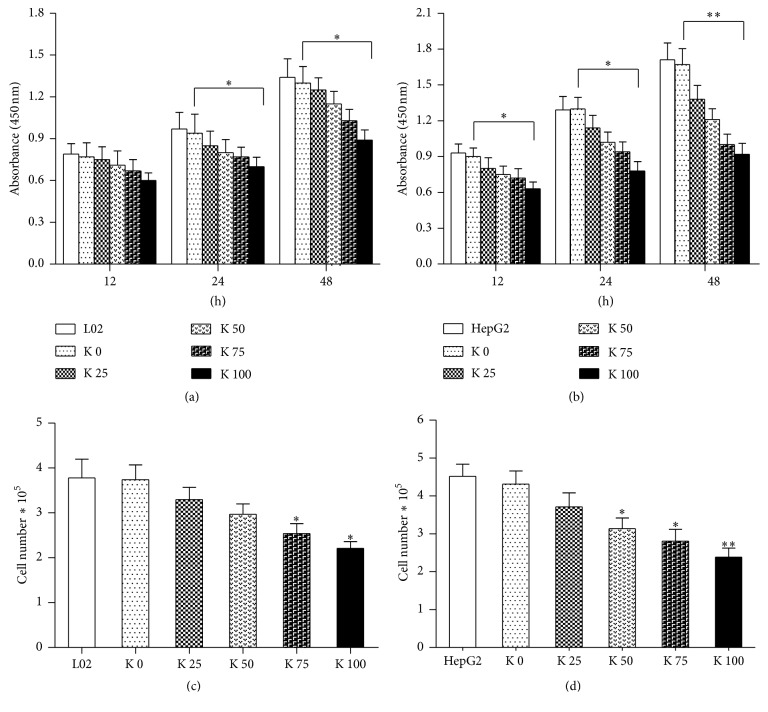
Potassium ions inhibited proliferation and growth of liver cells. L02 cells (3 × 10^3^) and HepG2 cells (3 × 10^3^) were added to 96-well plates cocultured with different concentrations of potassium ions and cultured at different time points (12, 24, and 48 hrs), respectively. We conduct the CCK-8 assay to assess how the potassium ions affected proliferation of L02 and HepG2 cells. (a) The absorbance of L02 cells decreased significantly at 24 hrs and 48 hrs (*P* < 0.05) after culture with potassium ions. (b) The absorbance value of HepG2 cells decreased significantly at 12 hrs and 24 hrs (*P* < 0.05) after being cultured with potassium ions and especially for 48 hrs (*P* < 0.01). L02 (3 × 10^5^) and HepG2 cells (3 × 10^5^) were added to 6-well plates cocultured with different concentrations of potassium ions and cultured for 48 hrs. (c) The cells number of L02 treated with potassium ions decreased after being cultured for 48 hrs (*P* < 0.05). (d) The cells number of HepG2 treated with potassium ions decreased significantly after culture for 48 hrs (*P* < 0.01). All data are represented as mean ± SEM. ^*∗*^ *P* < 0.05; ^*∗∗*^ *P* < 0.01.

To examine the effects of potassium ions on cell proliferation, cells were treated with increasing concentrations of potassium for indicated time points. By the CCK-8 assay, the results showed that potassium ions could inhibit the proliferation of L02 (Figure 1(a)) and HepG2 cells (Figure 1(b)), especially for HepG2 cells. The inhibition was both time and dose dependent. The proliferation of L02 cells cocultured with potassium ions decreased obviously after culture for 48 hrs (*P* < 0.05). The proliferation of HepG2 cells cocultured with potassium ions decreased especially at 48 hrs.

On the other hand, cell growth was quantified with total cell count. L02 and HepG2 cells were added to 6-well plates treated by the above methods and cultured for 48 hrs. As shown in Figure 1, the cell count for L02 (Figure 1(c)) and HepG2 (Figure 1(d)) was low with increasing concentration of potassium ions. The decreasing trend is more obvious for HepG2 than L02 cells.

### 3.2. Potassium Ions Affected the Cells Cycle of L02 and HepG2

**Figure 2:**
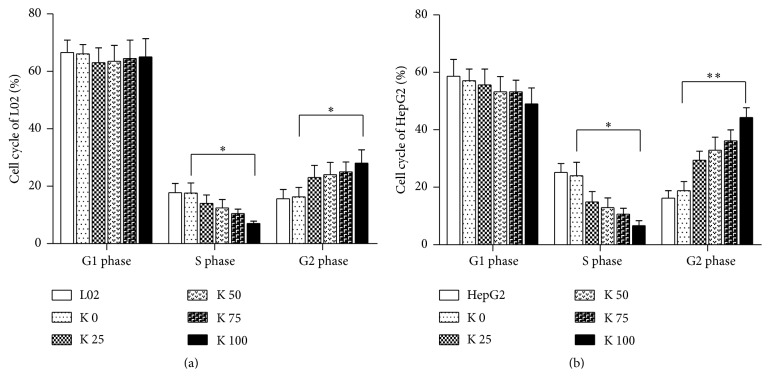
Potassium ions affected cell cycle of liver cells. L02 (3 × 10^5^) and HepG2 (3 × 10^5^) cells were added to 6-well plates cocultured with different concentrations of potassium ions for 48 hrs. Then the effects of potassium ions on cell cycle of L02 and HepG2 were determined by flow cytometry. (a) In L02 cells, potassium ions induced significantly decrease of S phase and increase of G2 phase in a dose-dependent manner (*P* < 0.05). (b) In HepG2 cells, potassium ions induced significantly decrease of S phase and increase of G1 phase in a dose-dependent manner (*P* < 0.01). All data are represented as mean ± SEM. ^*∗*^ *P* < 0.05, ^*∗∗*^ *P* < 0.01.

To test the effects of potassium ions on the cell cycle of L02 and HepG2 cells, we conducted flow cytometry assay. The results indicated that the effects of potassium ions on cell cycle of L02 cells (Figure 2(a)) and HepG2 cells (Figure 2(b)) were dose dependent. The cell number of the S phase in L02 and HepG2 cells both decreased, while it increased significantly in G2/M phase. These results demonstrated that potassium ions could arrest cell cycle at S phase and suppress the growth of L02 and HepG2 by preventing proper DNA replication. And the arrest is more significant for HepG2 than L02 cells.

### 3.3. Potassium Ion Induces Apoptosis of L02 and HepG2 Cells through Regulating Antiapoptotic Bcl-2 Family Members and Mitochondrial Proteins and Potassium Channel Related Protein

**Figure 3:**
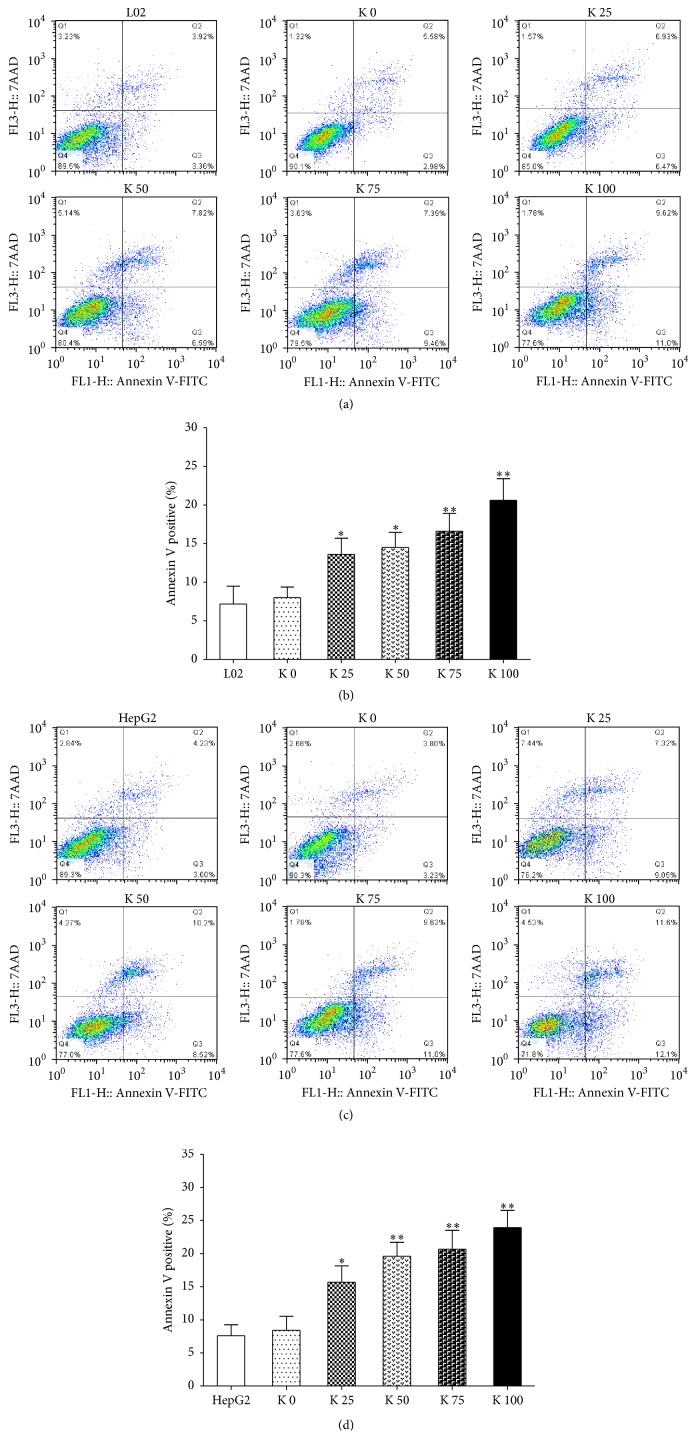
Potassium ions promoted cell apoptosis of liver cells. L02 (3 × 10^5^) and HepG2 (3 × 10^5^) cells were added to 6-well plates and cocultured with different concentrations of potassium ions for 48 hrs. Then, the effects of potassium ions on L02 and HepG2 cell apoptosis were determined by flow cytometry. (a, b) The apoptotic L02 cells treated with potassium ions increased significantly at 48 hrs (*P* < 0.05). (c, d) The apoptotic HepG2 cells treated with potassium ions increased significantly at 48 hrs, too (*P* < 0.01). All data are represented as mean ± SEM. ^*∗*^ *P* < 0.05, ^*∗∗*^ *P* < 0.01.

After being cocultured with potassium ions for 48 hrs, Annexin V-FITC/7-AAD staining was performed. And the numbers of Annexin V positive cells increased significantly. The results showed that the Annexin V positive cells of L02 (Figures 3(a) and 3(b)) and HepG2 (Figures 3(c) and 3(d)) both increased in a concentration dependence manner, especially for HepG2 cells.

**Figure 4:**
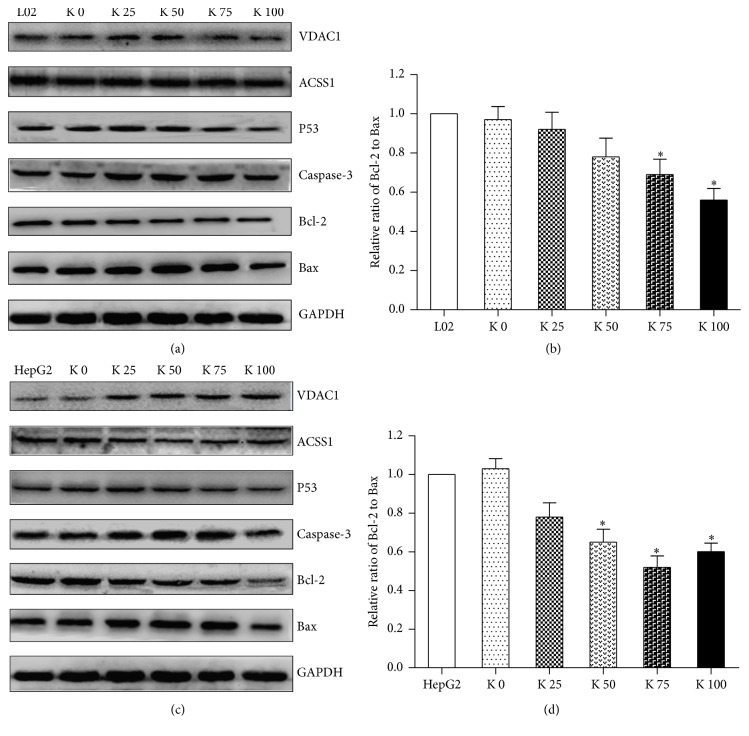
Potassium ions affected expression of apoptosis-related proteins and mitochondrial proteins of liver cells. The expression levels of P53, apoptosis-related proteins Bax, Bcl-2, caspase-3, or mitochondrial protein VDAC1, ACSS1, and K^+^ channel protein HERG in liver cells were determined by Western Blotting. (a) In L02 cells, the levels of Bax, caspase-3, VDAC1, and HERG increased after being cocultured with different concentrations of potassium ions in a dose-dependent manner, and the level of Bcl-2 decreased in a dose-dependent manner. The expression level of ACSS1 has no obvious change. (b) The Bcl-2/Bax ratios in L02 cells (*P* < 0.05) showed as relative optical density values of the protein bands normalized to GAPDH decreased in a dose-dependent manner. (c) In HepG2 cells, the expression levels of Bax, caspase-3, VDAC1, and HERG increased after being cocultured with different concentrations of potassium ions in a dose-dependent manner, and the expression level of Bcl-2 and ACSS1 decreased in a dose-dependent manner. Both the increasing and decreasing trends were more obvious than those in L02 cells. (d) The Bcl-2/Bax ratios in HepG2 cells (*P* < 0.05) showed as relative optical densities of the protein bands normalized to GAPDH decreased in a dose-dependent manner and more obviously than in L02 cells. All data are represented as mean ± SEM. ^*∗*^ *P* < 0.05.

As shown in Figure 4(a), the expression level of Bcl-2 downregulated in L02 cells, but Bax and caspase-3 upregulated. We could see the same trends in HepG2 cells (Figure 4(c)), but the trends are more obvious than those in L02 cell. Moreover, in K 100 group, the expression levels of Bax and caspase-3 downregulated. We determined the Bcl-2/Bax ratio in L02 (Figure 4(b)) and HepG2 cells (Figure 4(d)) at the protein level, which increased significantly in a concentration dependence manner, especially for HepG2 cells. They may be responsible for the apoptosis induced by potassium ions observed in L02 and HepG2 cells.

**Figure 5:**
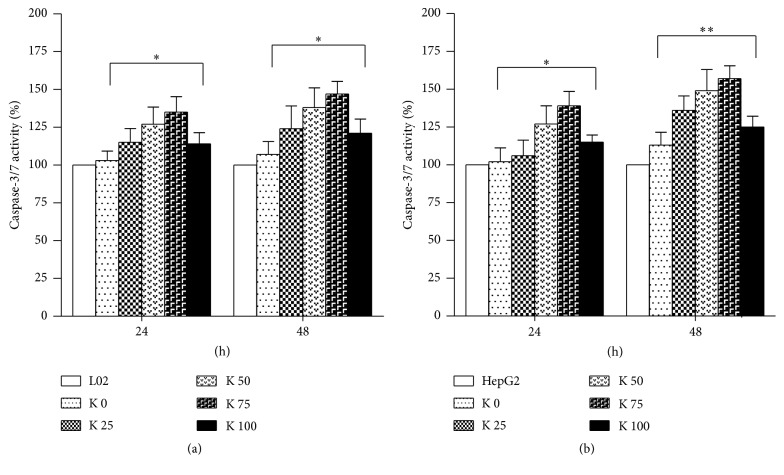
Potassium ions induced activation of caspase-3/7 activity in liver cells. (a) Potassium ions induced activation of caspase-3/7 activity in L02 cells. (b) Potassium ions induced activation of caspase-3/7 activity in HepG2 cells, too. All data are represented as mean ± SEM. ^*∗*^ *P* < 0.05, ^*∗∗*^ *P* < 0.01.

We performed caspase-3/7 activation detection to identify the apoptotic role of potassium ions on L02 and HepG2 cells. Potassium ions induced obvious activation of caspase-3/7 activity in L02 (Figure 5(a)) and HepG2 cells (Figure 5(b)). In summary, the findings demonstrated that potassium ions could reduce cell viability, promote cell apoptosis, and increase caspase-3/7 activity of L02 and HepG2 cells. This is more obvious in HepG2 cells than L02 cells.

In order to assess mitochondrial and potassium channel related genes expression changes, levels of mitochondria related proteins, VDAC1 and ACSS1, and potassium channel related protein HERG were evaluated after treatment of liver cells with potassium ions. As shown in Figures 4(a) and 4(c), the expression of ACSS1 downregulated in a dose-dependent manner, and the expression of VDAC1 and HERG upregulated, especially for HepG2 cells.

### 3.4. Potassium Ions Depolarized the Mitochondrial Membrane of L02 and HepG2 Cells

**Figure 6:**
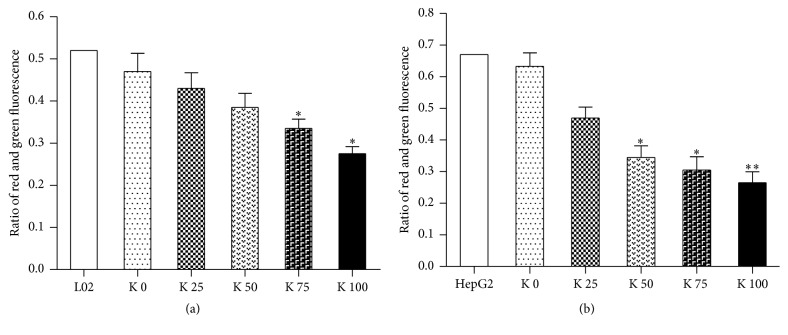
Potassium ions depolarized the mitochondrial membrane potential in liver cells. We measured mitochondrial membrane polarization in L02 and HepG2 cells at 48 hrs using JC-10. The ratio of red to green fluorescence decreased significantly in both L02 and HepG2 cells treated with potassium ions compared to control. (a) In L02 cells, the ratios of red to green fluorescence decreased in a dose-dependent manner (*P* < 0.05). (b) In HepG2, the ratios decreased in a dose-dependent manner cells and more obviously than in L02 cell (*P* < 0.05). All data are represented as mean ± SEM. ^*∗*^ *P* < 0.05, ^*∗∗*^ *P* < 0.01.

We used JC-10 to measure the mitochondrial membrane polarization in L02 and HepG2 cells. The results showed that the ratios of red to green fluorescence were significantly lower in potassium ions treated L02 cells (Figure 6(a)) and HepG2 cells (Figure 6(b)). And the results demonstrated mitochondrial membrane potential depolarized after exposure to potassium ions in liver cells. Moreover, these changes indicated mitochondrial membrane potential of liver cells depolarized in a concentration dependence manner after exposure to potassium ions, especially for HepG2 cells.

## 4. Discussions

Hepatocellular carcinoma (HCC) is a common malignant tumor of liver, usually in individuals with developing chronic liver disease or cirrhosis. HCC is the common cause of cancer and is ranked fifth while it is also the second commonest cause of cancer deaths among human [11]. Patients with HCC have poor prognosis; thus urgent measures to curb HCC are needed [12–14].

The hallmarks of tumor contain the inhibition of programmed cell death and abnormal cell proliferation [2]. Exploring new strategies to induce apoptosis and inhibit proliferation of tumor cells is needed. Researchers found that the first features of potassium channels in nonneural cells were the role of proliferation [1, 2]. Our research also showed that potassium ions could inhibit proliferation of liver cells, especially for HepG2 cells. Moreover, the results of cells cycle analysis indicated that potassium ions could block the S phase of the cell cycle and suppress the growth of L02 and HepG2 cells through preventing proper DNA replication. The antitumor influence of potassium ions has been proved in most human tumor cells through regulation of cell cycle and apoptosis [15, 16]. Inhibition of apoptosis can result from carcinogenesis, so induction of apoptosis may be a better potential antitumor therapeutic strategy [17–20]. However, effects of potassium ions on hepatocellular carcinoma have not been reported. Our results identified that potassium ions promoted cell apoptosis and induced obvious activation of caspase-3/7 activity in both L02 and HepG2 cells, especially for HegG2 cells. Therefore, the specific mechanisms need to be deeply investigated.

Apoptosis is an important mechanism for eliminating both excess normal cells and those cells which have sustained damage. The two common pathways of apoptosis are exogenous (death receptor mediated) and intrinsic (mitochondrial) approach [21, 22]. Potassium channel is the important factor for the changes in membrane potential during the cell-cycle progression. Therefore, blocking the activity of potassium channels could induce antiproliferation effects [23, 24]. Concomitantly, at the initiation of apoptosis, intracellular potassium ions concentration decreased. Potassium channel is a key channel to maintain the stability of cell membrane potential. Because there are many types of these channels mediating dominant potassium efflux, they play a significant role in membrane permeability and cell volume regulation [25, 26]. The tumor cells with negative resting potential are usually smaller than normal cells [27]; thus, inflow of more potassium ions through high level expression of potassium channels in response to the certain physical process is possible. Our results demonstrated that the mitochondrial pathway of apoptosis is important. After being treated with potassium ions for 48 hrs, mitochondrion membrane potential (ΔΨm) of HepG2 and L02 cells both decreased, which explained potassium ions induced apoptosis of HepG2 and L02 cells. Potassium ions may inhibit cell-cycle progression and proliferation through reducing the production of ATP and mitochondrial membrane depolarization.

In addition to supplying cellular energy, mitochondria are involved in other tasks, such as signaling, cellular differentiation, and cell death, as well as maintaining control of cell cycle and cell growth. Mitochondrion is an important factor controlling the intrinsic pathway of cell apoptosis, including release of caspase cofactors, such as cytochrome c (Cyt c) and SMAC, generation of apoptotic body, and induction of apoptosis. Furthermore, the Bcl-2 gene family is a key point of the mitochondria pathway [28]. This pathway involves several members including Bax, a proapoptotic protein, and Bcl-2, an antiapoptotic protein, which played a crucial role in regulation of apoptosis [29, 30]. The Bcl-2/Bax ratio has been used to evaluate the cell apoptosis, and its reduction could activate the expression of caspase proteins [31, 32]. By Western Blot detection, we found that Bcl-2/Bax ratio in liver cells decreased after being cocultured with potassium ions for 48 hrs. These data showed that the decrease of the ratio is closely related to liver cells apoptosis induced by potassium ions.

Voltage-dependent anion-selective channel protein 1 (VDAC1) is a voltage-dependent anion channel which is involved in the regulation of cell metabolism, mitochondrial apoptosis, and spermatogenesis. VDAC1, mitochondrial porin 1, played a pivotal role in mitochondria mediated apoptosis and transporting various ions or small molecules across the outer mitochondrial membrane. In particular, VDAC1 is the major ion transport channel and is implicated in cancer. The increasing expression of VDACs may be a specific target for treatment of cancer [33]. HK2 binding to VDAC antagonizes cell apoptosis through inhibition of Bax-induced releasing of Cyt c [7, 34, 35] and inhibits the mitochondrial permeability transition [36]. The decomposition product of HK seems to destroy aerobic glycolysis and energy balance of cells, regulate the interaction of Bcl-2 protein family, and promote mitochondrial VDAC oligomer formation to induce cell death [37, 38]. Thus, the HK-VDAC complex has become an important target for treatment of cancer [39, 40]. Our results showed that potassium ions upregulated the expression of VDAC1 in a dose-dependent manner. Therefore, potassium ions overbalanced the mitochondrial membrane potential through upregulating VDAC1 or breaking the balance of Bcl-2/Bax ratio and then induced Cyt c released from mitochondria, caspase activation, and caspase-3/7 ration imbalance, finally resulting in cell apoptosis.

Another protein ACSS1 was detected, which is the key protein in mitochondrial respiratory chains and encodes a mitochondrial acetyl-CoA synthetase that it used to produce ATP molecules. Our results indicated that potassium ions induced downregulation of ACSS1, and then mitochondrial energy metabolism was restrained. It caused a reduction of cell proliferation and cell-cycle arrest. Both the mitochondrial membrane depolarization and metabolic disorders are the suppressor of cell cycle. The expression of ACSS1 in HepG2 cells downregulated after being cocultured with potassium ions, but there was no difference in L02 cells. It demonstrated that potassium ions could destroy the mitochondrial respiratory chains and restrain the mitochondrial energy metabolism. Moreover, for the differences between L02 and HepG2 cell, ACSS1 may play a crucial role.

In order to further evaluate the gene expressions change related to potassium channel induced by potassium ions in L02 and HepG2 cells, the expression of HERG was evaluated. HERG is a gene (KCNH2) that encodes a protein known as Kv11.1, the alpha subunit of a potassium ions channel [41]. HERG involves regulation of nervous system functions and also carcinogenesis and development of leukemia tumor [41]. The expression levels of HERG in liver cells upregulated, especially for HepG2 cells. The results indicated that these biological functions affected by potassium ions were associated with channel protein HERG.

It is known that potassium ions play an extensive role. We studied the biological function induced by potassium ions in liver cells and explored their molecular mechanism. By facilitating expression of channel protein HERG, potassium ions may prevent cells from being shunted to procancerous pathways and overbalance the mitochondrial membrane potential through upregulating expression of VDAC1 or breaking the balance of Bcl-2/Bax ratio and then induced cytochrome c released from mitochondria, caspase activation, and caspase-3/7 ration imbalance, finally resulting in cell apoptosis.

In conclusion, our results demonstrated that potassium ions may be a key regulator of liver cell function. Potassium ions could inhibit tumorigenesis through inducing apoptosis of hepatoma cells by upregulating potassium ions transport channel proteins HERG and VDAC1.
